# The shared microbiota of humans and companion animals as evaluated from *Staphylococcus* carriage sites

**DOI:** 10.1186/s40168-014-0052-7

**Published:** 2015-01-23

**Authors:** Ana M Misic, Meghan F Davis, Amanda S Tyldsley, Brendan P Hodkinson, Pam Tolomeo, Baofeng Hu, Irving Nachamkin, Ebbing Lautenbach, Daniel O Morris, Elizabeth A Grice

**Affiliations:** Department of Dermatology, Perelman School of Medicine, University of Pennsylvania, 421 Curie Blvd, 1007 Biomedical Research Building, Philadelphia, PA 19104 USA; Department of Environmental Health Sciences, Johns Hopkins Bloomberg School of Public Health, Baltimore, MD USA; Department of Biostatistics and Epidemiology, Perelman School of Medicine, University of Pennsylvania, Philadelphia, PA USA; Department of Pathology and Laboratory Medicine, Perelman School of Medicine, University of Pennsylvania, Philadelphia, PA USA; Department of Medicine, Perelman School of Medicine, University of Pennsylvania, Philadelphia, PA USA; Department of Clinical Studies, School of Veterinary Medicine, University of Pennsylvania, Philadelphia, PA USA

**Keywords:** 16S rRNA, Methicillin-resistant *Staphylococcus aureus* (MRSA), Microbiome, Pet, *Staphylococcus*, Skin and soft tissue infection (SSTI)

## Abstract

**Background:**

*Staphylococcus aureus* and other coagulase-positive staphylococci (CPS) colonize skin and mucous membrane sites and can cause skin and soft tissue infections (SSTIs) in humans and animals. Factors modulating methicillin-resistant *S. aureus* (MRSA) colonization and infection in humans remain unclear, including the role of the greater microbial community and environmental factors such as contact with companion animals. In the context of a parent study evaluating the households of outpatients with community MRSA SSTI, the objectives of this study were 1) to characterize the microbiota that colonizes typical coagulase-positive *Staphylococcus* spp. carriage sites in humans and their companion pets, 2) to analyze associations between *Staphylococcus* infection and carriage and the composition and diversity of microbial communities, and 3) to analyze factors that influence sharing of microbiota between pets and humans.

**Results:**

We enrolled 25 households containing 56 pets and 30 humans. Sampling locations were matched to anatomical sites cultured by the parent study for MRSA and other CPS. Bacterial microbiota were characterized by sequencing of 16S ribosomal RNA genes. Household membership was strongly associated with microbial communities, in both humans and pets. Pets were colonized with a greater relative abundance of Proteobacteria, whereas people were colonized with greater relative abundances of Firmicutes and Actinobacteria. We did not detect differences in microbiota associated with MRSA SSTI, or carriage of MRSA, *S. aureus* or CPS. Humans in households without pets were more similar to each other than humans in pet-owning households, suggesting that companion animals may play a role in microbial transfer. We examined changes in microbiota over a 3-month time period and found that pet staphylococcal carriage sites were more stable than human carriage sites.

**Conclusions:**

We characterized and identified patterns of microbiota sharing and stability between humans and companion animals. While we did not detect associations with MRSA SSTI, or carriage of MRSA, *S. aureus* or CPS in this small sample size, larger studies are warranted to fully explore how microbial communities may be associated with and contribute to MRSA and/or CPS colonization, infection, and recurrence.

**Electronic supplementary material:**

The online version of this article (doi:10.1186/s40168-014-0052-7) contains supplementary material, which is available to authorized users.

## Background

Methicillin-resistant *Staphylococcus aureus* (MRSA) is a major cause of skin and soft tissue infections (SSTI), with 80,000 invasive MRSA SSTI and 11,000 resultant deaths reported in the United States in 2011 [1]. Rates of asymptomatic nasal colonization in the general public range from 1.5% for MRSA to ≥30% for *S. aureus* at any given time [2,3]. Pets, including cats, dogs, horses, and exotic species, may carry methicillin-susceptible *S. aureus* (MSSA) and MRSA but more commonly are colonized by other coagulase-positive staphylococci (CPS), particularly *S. pseudintermedius* and *S. schleiferi*, which can also carry genes conferring resistance to β-lactam antimicrobials and cause SSTI [4-6].

The natural reservoir of *S. aureus* is the anterior nares as well as other anatomical skin sites in humans, particularly the axillae and groin [7]. In animals, the nares, mouth, and perineum are typical CPS carriage sites [8]. The skin, a common site of staphylococcal infection in both humans and animals, is the body’s interface with the external environment and an ecosystem harboring diverse microbial populations that provide important functions to the host. Vital functions of the skin’s microbiome include provision of a blockade to opportunistic and pathogenic microorganisms, and contributions to the regulation of immunity and inflammation [9-11]. Thus, the skin microbiota may modulate cutaneous infection and disease, such as SSTI caused by staphylococci.

The role of microbiota in modulating resistance to pathogenic and opportunistic microorganisms and in regulating the host immune response is increasingly evident; furthermore, pathogens function in the context of microbial communities. For example, the skin commensal *S. epidermidis* can inhibit *S. aureus* colonization and biofilm formation in the nares [12]. *S. epidermidis* can also act as a reservoir of antibiotic-resistance genes and other genetic elements that are readily transferred to *S. aureus* via horizontal gene transfer [13]. These potentially counter-productive functions illustrate the complexity of microbe-microbe interactions as they relate to *S. aureus* colonization and infection.

The primary mode of MRSA transmission is through direct contact, for example with a colonized or infected individual [14,15]. Even with antibiotic treatment and decolonization protocols, MRSA may continue to be carried asymptomatically by humans [16,17]. Indeed, recurrent MRSA infection has been reported in up to 31% of those affected within 6 months of the first SSTI [18]. Companion animals and other household factors may play a role in MRSA SSTI recurrence [19-21]. Recent findings that dog ownership increases the degree of shared skin microbiota within households underscores the potential role that pets may play in sharing of microbiota [22]. Therefore, studying human and companion animal populations in parallel may provide greater insight into the dynamics governing CPS-microbiota-host interactions.

We hypothesized that microbial communities, particularly at anatomical sites associated with staphylococcal carriage, may play a role in MRSA (or other CPS) colonization, carriage, infection, and recurrence. We further hypothesized that companion animals living in the household may participate in sharing of microbiota. To test these hypotheses, we used culture-independent sequencing of the bacterial 16S ribosomal RNA (rRNA) gene to characterize microbiota colonizing humans and their companion animals in households. Sampling of subjects was conducted in the context of a larger study which enrolled household members and pets of index patients diagnosed with an MRSA SSTI. Our objectives were to 1) characterize the microbiota colonizing anatomical sites of staphylococci carriage in humans and their companion animals, 2) analyze associations between MRSA and CPS infection and carriage and the composition and diversity of the microbial community, and 3) analyze factors that influence sharing of microbiota between humans and companion animals.

## Results

### Enrollment

#### Baseline sampling

Twenty-five households were enrolled and sampled, representing 78% of homes enrolled between July and December 2012 in the parent study. Six households without pets were excluded due to pending IRB oversight at the time of the study visit; one household with a single dog was excluded from animal sampling due to dog aggression (humans were not eligible to be sampled at that study visit). Humans were sampled from seven households, of which four (57%) had pets. Eighteen households were enrolled from which only pets participated in the study. Households in which people were sampled were similar in terms of the number of human household members and number of companion animals in the household compared to households in which only pets were sampled.

#### Three-month follow-up sampling

Twenty households (80%) remained in the study and participated in the follow-up home study visit 3 months after the baseline study visit. This study visit followed a 1-week treatment of people with nasal mupirocin ointment and chlorhexidine body washes in the 12 homes randomized to receive it. Pets were not treated.

Only cats and dogs were included in our analyses of pets. Samples from other mammalian pets were collected but not included in the analyses due to small sample size (four hamsters, one ferret, one rabbit, and one sugar glider). All of these “pocket pet” species were resident in homes in which dogs and/or cats also were present.

### Subjects and characterization of microbiota colonizing sites of staphylococcal carriage

Characteristics of households and study participants (human and companion animal) according to visit are provided in Table 1, which demonstrates that human subjects were of working age or younger (≤65 years old), and companion animals ranged in age from two to 135 months (≤12 years old). From 288 samples (34 cat nares, 34 cat mouths, 53 dog nares, 55 dog mouths, 51 human nares, 51 human axillae/groin, and 10 human lesion site samples), 11,474,465 sequences were obtained and analyzed. Sequence counts stratified by host species and anatomical site are shown in Additional file 1: Table S1. The taxonomic relative abundance data for the “pocket pets” are illustrated in Additional file 2: Figure S1.

**Table 1:** **Study demographics**

	**Total sampled**	**Humans only**	**Humans and pets**	**Pets only**
Baseline study visit				
Households				
Number of households	25	3	4	18
Number of people, median (range)	4 (1–8)	4 (2–5)	5 (4–7)	4 (1–8)
Number of pets, median (range)	2 (0–13)	0	2 (1–10)	2 (1–13)
Human subjects				
Index subjects	6	3	3	-
Female:male	3:3	2:1	1:2	-
Age in years, median (range)	8.5 (2–48)	5 (2–48)	13 (6–15)	-
CPS + at any site, *n* = 4	3	1	2	
*S. aureus* + at any site, *n* = 4	1	1	0	
MRSA + at any site	1	1	0	-
Non-index subjects	24	7	17	-
Female:male	12:12	4:3	8:9	-
Age in years, median (range)	25 (3–54)	23 (9–42)	26 (3–54)	-
CPS + at any site, *n* = 18	15	1	14	
*S. aureus* + at any site, *n* = 18	3	0	3	
MRSA + at any site	3	0	3	-
Pet subjects				
Dogs	36	-	5	31
Female:male	21:15	-	3:2	18:13
Age in months, median (range)	24 (2–132)	-	24 (6–120)	24 (2–132)
Neutered:unneutered	9:27	-	1:4	8:23
CPS + at any site	34	-	4	30
*S. aureus* + at any site	6	-	1	5
MRSA + at any site	2	-	2	0
Cats	20	-	3	17
Female:male	11:9	-	3:0	8:9
Age in months, median (range)	27 (4–135)	-	12 (8–20)	53 (4–135)
Neutered:unneutered	16:4	-	2:1	14:3
CPS + at any site	13	-	2	11
*S. aureus* + at any site	4	-	1	3
MRSA + at any site	2	-	2	0
Three-month follow-up study visit				
Households				
Number of households	20	2	3	15
Number randomized to treatment	12	2	2	8
Number of people, median (range)	4 (1–8)	4.5 (4–5)	5 (4–7)	4 (1–8)
Number of pets, median (range)	2 (0–10)	0	2 (1–8)	3 (1–10)
Human subjects				
Index subjects	4	2	2	-
Randomized to treatment	3	2	1	-
Female:male	1:3	1:1	0:2	-
Age in years, median (range)	9 (2–15)	3.5 (2–5)	14 (13–15)	-
CPS + at any site, *n* = 4	3	0	3	
*S. aureus* + at any site, *n* = 4	1	0	1	
MRSA + at any site	0	0	0	-
Non-index subjects	17	6	11	-
Randomized to treatment	14	6	8	
Female:male	8:9	3:3	5:6	-
Age in years, median (range)	26 (3–54)	21 (9–42)	26 (3–54)	-
CPS + at any site, *n* = 14	11	3	8	
*S. aureus* + at any site, *n* = 14	4	1	3	
MRSA + at any site	0	0	0	-
Pet subjects				
Dogs	19	-	4	15
Female:male	12:7	-	2:2	10:5
Age in months, median (range)	37 (6–132)	-	42 (6–120)	37 (9–132)
Neutered:unneutered	7:12	-	1:3	6:9
CPS + at any site	17	-	3	14
*S. aureus* + at any site	4		0	4
MRSA + at any site	3	-	0	3
Cats	14	-	2	12
Female:male	8:6	-	2:0	6:6
Age in months, median (range)	15 (4–135)	-	10 (8–12)	23 (4–135)
Neutered:unneutered	10:4	-	1:1	9:3
CPS + at any site	11	-	1	10
*S. aureus* + at any site	4		0	4
MRSA + at any site	1	-	0	1

For pet-only households, humans were present, but were not eligible for participation. Data from the 3-month follow-up visit are limited to people and pets sampled longitudinally, excluding new household members present at the 3-month visit.

**Figure 1:**
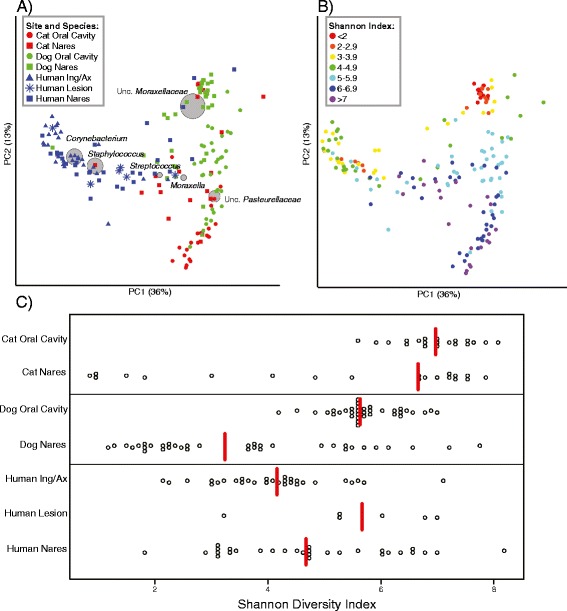
**Distinct microbiomes colonize staphylococcal carriage sites of dogs, cats, and humans. (A)** Weighted UniFrac metric principal coordinates analysis (PCoA) comparing the microbiomes of cat oral cavities (red circles), cat nares (red squares), dog oral cavities (green circles), dog nares (green squares), human inguinal creases/axillae (blue triangles), human nares (blue squares), and human healing lesions (blue stars). The grey circles represent the six most abundant genus-level consensus taxonomies (unclassified *Moraxellaceae*, *Corynebacterium*, *Staphylococcus*, unclassified *Pasteurellaceae*, *Moraxella*, and *Streptococcus*) contributing to variation, and size of spheres indicates relative abundance. Percent variation explained by PC1 and PC2 is represented in parentheses by each axis. **(B)** Alpha diversity characteristics of pet and human *Staphylococcus* carriage sites, visualized by the weighted UniFrac metric PCoA comparing microbial communities and alpha diversity (Shannon diversity index) overlaid on the same PCoA plot. **(C)** Shannon diversity index values comparing cat, dog, and human staphylococcal carriage sites are depicted with each circle representing one sample and medians indicated by red lines.

To obtain an overall view of pet microbiota and how they compare to humans at anatomical sites of staphylococcal carriage sampled in the parent study, we calculated the weighted UniFrac metric to infer distances between communities and visualized all samples using principal coordinates analysis (PCoA) (Figure 1A). Host species and anatomical site had a strong, significant effect on microbial community composition (*R* = 0.58; weighted UniFrac; *p* < 0.001; Table 2). The bacterial genus-level taxa contributing to the clustering, and representing 57.1% of the sequences, are represented by grey circles in Figure 1A. Because not all sampled anatomical sites were consistent across all host species, we specifically compared nasal microbiota to examine differences between cats, dogs, and humans, as all host species were sampled from the nares. Again, host species had a significant effect on the nares microbiota (*R* = 0.43; weighted UniFrac; *p* < 0.001; Table 2). *Bacillales* and *Actinomycetes* cluster with human nasal microbiota and include the genera *Corynebacterium* and *Staphylococcus*, consistent with previous studies of the nasal microbiota of healthy humans [23,24]. Cat and dog nasal microbiota clustered with Gram-negative Proteobacteria such as *Moraxella* and unclassified *Pasteurellaceae*.

**Table 2:** **Association of different factors with microbial communities at staphylococcal carriage sites at the baseline visit**

**Comparison category**	**Host species**	**Anatomic site**	**Beta diversity metric**	***R*** **statistic and significance**
Host species and anatomical site (*cat*, *dog*, *or human and nares*, *oral cavity or inguinal/axilla*)	All	All	Weighted UniFrac	0.5753***
			Unweighted UniFrac	0.4731***
			Bray-Curtis	0.5803***
			Binary Jaccard	0.5470***
Host species (*cat*, *dog*, *or human*)	All	Nares	Weighted UniFrac	0.4326***
			Unweighted UniFrac	0.2525***
			Bray-Curtis	0.5230***
			Binary Jaccard	0.5378***
Household membership	Cats	Nares	Weighted UniFrac	0.4698*
			Unweighted UniFrac	0.7501**
			Bray-Curtis	0.7776**
			Binary Jaccard	0.9451**
	Cats	Oral cavity	Weighted UniFrac	0.5246**
			Unweighted UniFrac	0.5815**
			Bray-Curtis	0.5714**
			Binary Jaccard	0.6018**
	Dogs	Nares	Weighted UniFrac	0.1786
			Unweighted UniFrac	0.4100*
			Bray-Curtis	0.0363
			Binary Jaccard	0.4170*
	Dogs	Oral cavity	Weighted UniFrac	0.4090**
			Unweighted UniFrac	0.3042**
			Bray-Curtis	0.4372**
			Binary Jaccard	0.5328**
	Humans	Nares	Weighted UniFrac	0.2525*
			Unweighted UniFrac	0.4926**
			Bray-Curtis	0.3203**
			Binary Jaccard	0.5012**
	Humans	Inguinal/axilla	Weighted UniFrac	−0.0178
			Unweighted UniFrac	0.1196
			Bray-Curtis	0.0472
			Binary Jaccard	0.1931*
Index subject status (*Yes or no*)	Humans	Nares	Weighted UniFrac	0.0558
			Unweighted UniFrac	−0.0774
			Bray-Curtis	0.0807
			Binary-Jaccard	−0.1209
	Humans	Inguinal/axilla	Weighted UniFrac	0.1084
			Unweighted UniFrac	−0.1137
			Bray-Curtis	0.1399
			Binary Jaccard	0.1010
Household pet ownership (*Yes or no*)	Human	Nares	Weighted UniFrac	0.1466
			Unweighted UniFrac	−0.0579
			Bray-Curtis	0.0507
			Binary Jaccard	−0.0350
	Human	Inguinal/axilla	Weighted UniFrac	−0.0256
			Unweighted UniFrac	0.0495
			Bray-Curtis	−0.0319
			Binary Jaccard	0.1119

Summarized are ANOSIM analyses assessing association of various metadata on microbial community distances measured using weighted and unweighted UniFrac, Bray-Curtis index, and Jaccard index. *p < 0.05, **p < 0.005, and ***p ≤ 0.001 (FDR adjusted values).

**Figure 2:**
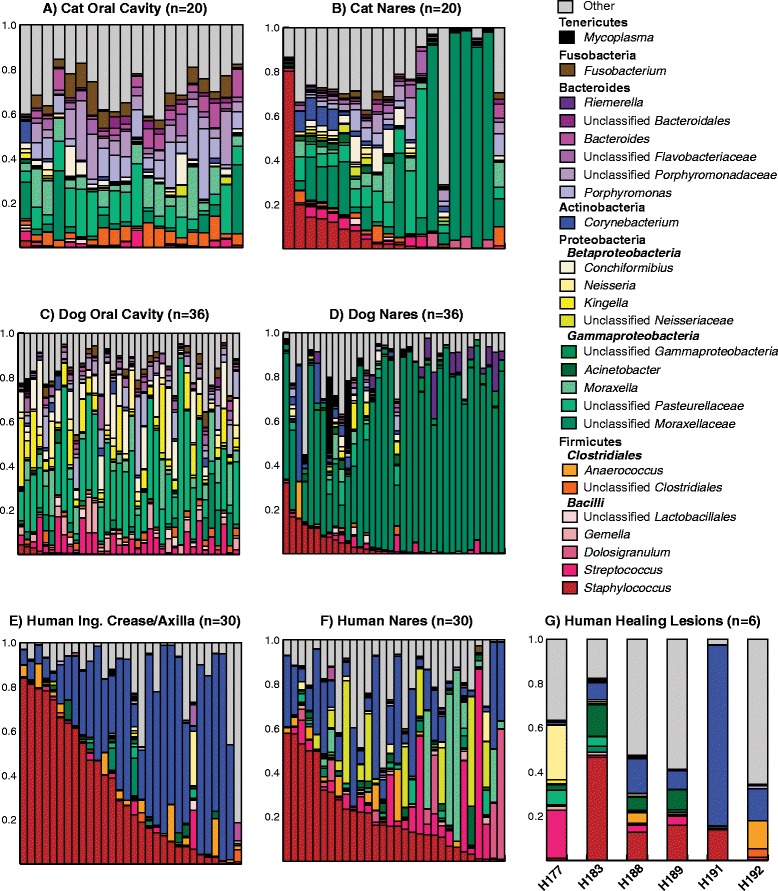
**Taxonomic classification of microbiota colonizing pet and human sites of Staphylococcus carriage and lesions.** Bacterial relative abundance of **(A)** cat oral cavity, **(B)** cat nares, **(C)** dog oral cavity, **(D)** dog nares, **(E)** human combined inguinal crease/axillae, **(F)** human nares, and **(G)** human healing lesions. The genera are colored by their phylum membership: Proteobacteria: green (Gammaproteobacteria) and yellow (Betaproteobacteria); Firmicutes: red/pink (Bacilli) and orange (Clostridia); Actinobacteria: blue; Bacteroidetes: purple; Fusobacteria: brown; Tenericutes: black; all others: grey).

These taxonomic differences in cat and dog microbiota, compared to human microbiota, are underscored when examining relative abundance even at the phylum level. In cats and dogs, Proteobacteria is the dominant bacterial phylum, followed by Bacteroidetes and Firmicutes (Figure 2A–D). Less abundant phyla include Fusobacteria, Spirochaetes, Tenericutes, and SR1 (Additional file 3: Table S2). In humans, Firmicutes is the dominant phylum, followed by Actinobacteria in the skin inguinal crease and axillae sites, or Proteobacteria in the nares. At the genus level, unclassified *Moraxellaceae* and unclassified *Pasteurellaceae*, both of phylum Proteobacteria, were the two most abundant genera present in cats and dogs (Figure 2A–D). *Corynebacterium* and *Staphylococcus*, of phylum Actinobacteria and Firmicutes, respectively, were the most abundant genera in humans (Figure 2E–F). A complete genus-level relative abundance table of all samples is found in Additional file 3: Table S2. The short read lengths employed in this study precluded us from resolving species-level taxonomic assignments.

To quantify and compare alpha diversity, we calculated and compared the Shannon diversity index for all host species and sites. These index values were overlaid on a weighted UniFrac PCoA plot, illustrating the host species and site specificity of clustering (Figure 1B–C). The cat oral cavity contained the greatest alpha diversity (a median Shannon index value of 7.0), while the dog oral cavity had significantly lower alpha diversity in comparison (median Shannon index value of 5.6; *p* = 2.6 × 10^−8^; Figure 1C). In dogs, the oral cavity had significantly higher Shannon diversity index values than the nares (median Shannon index value of 3.2; *p* = 1.8 × 10^−7^).

To identify potential signature microbiota of host species and anatomical sites, we identified operational taxonomic units (OTUs) present in 90% and 100% of sample types and their respective relative abundances (Additional file 4: Table S3). Ninety percent of cat nares contained OTUs classified as *Staphylococcus*, which belonged to the same OTU that was present in 100% of human nares, inguinal/axilla, and lesion samples. Core OTUs of the cat nares only comprised 13.1% total relative abundance, whereas core OTUs of the cat oral cavity comprised 19.0% of total relative abundance and included many OTUs classified as anaerobes (*Porphyromonadaceae*, *Clostridiales*, and *Bacteroides*), *Streptococcus*, and various Gram-negative bacteria (*Pasteurella*, *Moraxella*, and *Kingella*). The dog oral cavity contained similar core OTUs as the cat oral cavity and comprised 29.1% of total bacterial relative abundance. Core OTUs of dog nares comprised 50.5% of total relative abundance and consisted of *Moraxellaceae*, *Staphylococcus*, and unclassified Gammaproteobacteria OTUs. Core OTUs of the human inguinal/axilla comprised 34.5% of bacterial relative abundance and included OTUs classified as *Staphylococcus*, *Corynebacterium*, and *Finegoldia*, whereas core OTUs of the nares comprised 18.1% of relative abundance and additionally included OTUs classified as *Streptococcus* and *Enhydrobacter*. Taken together, these observations indicate that composition and diversity of microbial communities are variable across anatomical sites of sampling and host species. We therefore analyzed anatomical sites individually (as opposed to collectively) when examining the microbiota with respect to MRSA and CPS infection and/or carriage and sharing in households.

### Microbial community composition in index subjects with MRSA SSTI

We examined the microbiota colonizing healing lesions caused by MRSA SSTI in the index subjects (*n* = 6) enrolled in the study. In addition to *Staphylococcus*, which was present at a median relative abundance of 13.25% (range: 0.25%–46.75%), the genus-level taxa found at the highest relative abundances in the lesions were (in descending order) *Corynebacterium*, *Acinetobacter*, unclassified bacteria, *Streptococcus*, *Pseudomonas*, *Prevotella*, *Micrococcus*, unclassified *Enterobacteriaceae*, and unclassified *Pasteurellaceae* (Figure 2G). All healing MRSA lesions contained OTUs classified as *Staphylococcus*, *Corynebacterium*, *Micrococcus*, and *Streptococcus* (Additional file 4: Table S3).

We compared the microbiota of index and non-index human subjects of the nares and the inguinal crease/axilla regions to determine if recent MRSA SSTI was associated with differences in microbial community composition at these carriage sites. Overall, no differences were detected using alpha- or beta-diversity metrics (Table 3), and no taxa were differentially represented. Together, these results indicate that recent MRSA SSTI may not be associated with differences in microbial community composition and diversity at staphylococcal carriage sites, or our sample size was not sufficient to detect significant differences. Alternatively, our findings could be a result of the initial treatment regimens prescribed by diagnosing physicians for the index subjects, given that only one of six index subjects was positive for MRSA carriage at the initial sampling visit.

**Table 3:** **Association of staphylococcal carriage with microbial communities at carriage sites at the baseline visit**

**Comparison category**	**Host species**	**Anatomic site**	**Beta diversity metric**	***R*** **statistic and significance**
CPS colonization status (*Yes or no*)	Humans	Nares	Weighted UniFrac	0.008
			Unweighted UniFrac	0.0389
			Bray-Curtis	−0.0165
			Binary Jaccard	0.0113
	Humans	Inguinal/axilla	Weighted UniFrac	0.1514
			Unweighted UniFrac	0.1391
			Bray-Curtis	0.2577
			Binary Jaccard	0.1983
	Cats	Nares	Weighted UniFrac	−0.0729
			Unweighted UniFrac	−0.0683
			Bray-Curtis	−0.0626
			Binary Jaccard	−0.0459
	Cats	Oral cavity	Weighted UniFrac	−0.0356
			Unweighted UniFrac	−0.0347
			Bray-Curtis	−0.0717
			Binary Jaccard	−0.0480
	Dogs	Nares	Weighted UniFrac	0.0994
			Unweighted UniFrac	0.0369
			Bray-Curtis	0.1299
			Binary Jaccard	0.1483
	Dogs	Oral cavity	Weighted UniFrac	0.1491
			Unweighted UniFrac	0.2495*
			Bray-Curtis	0.1265
			Binary Jaccard	0.1751
*S. aureus* colonization status (*Yes or no*)	Humans	Nares	Weighted UniFrac	−0.0063
			Unweighted UniFrac	−0.0938
			Bray-Curtis	−0.0845
			Binary Jaccard	−0.1714
	Humans	Inguinal/axilla	Weighted UniFrac	No *S. aureus* at this time point
			Unweighted UniFrac	
			Bray-Curtis	
			Binary Jaccard	
	Cats	Nares	Weighted UniFrac	−0.0228
			Unweighted UniFrac	−0.1451
			Bray-Curtis	−0.1280
			Binary Jaccard	−0.0980
	Cats	Oral cavity	Weighted UniFrac	−0.2021
			Unweighted UniFrac	−0.1815
			Bray-Curtis	−0.220
			Binary Jaccard	−0.1398
	Dogs	Nares	Weighted UniFrac	−0.0540
			Unweighted UniFrac	−0.1028
			Bray-Curtis	−0.1452
			Binary Jaccard	−0.2037
	Dogs	Oral cavity	Weighted UniFrac	−0.1012
			Unweighted UniFrac	−0.1176
			Bray-Curtis	−0.1831
			Binary Jaccard	−0.0311
MRSA colonization status (*Yes or no*)	Humans	Nares	Weighted UniFrac	−0.0236
			Unweighted UniFrac	−0.1443
			Bray-Curtis	−0.0718
			Binary Jaccard	−0.1910
	Humans	Inguinal/axilla	Weighted UniFrac	0.1150
			Unweighted UniFrac	−0.1172
			Bray-Curtis	0.1653
			Binary Jaccard	0.0008
	Cats	Nares	Weighted UniFrac	−0.2287
			Unweighted UniFrac	−0.2056
			Bray-Curtis	−0.2460
			Binary Jaccard	−0.2810
	Cats	Oral cavity	Weighted UniFrac	−0.2582
			Unweighted UniFrac	−0.0009
			Bray-Curtis	−0.2450
			Binary Jaccard	−0.0194
	Dogs	Nares	Weighted UniFrac	−0.2705
			Unweighted UniFrac	−0.0066
			Bray-Curtis	−0.2904
			Binary Jaccard	−0.2473
	Dogs	Oral cavity	Weighted UniFrac	No MRSA at this time point
			Unweighted UniFrac	
			Bray-Curtis	
			Binary Jaccard	

Summarized are ANOSIM analyses assessing association of various metadata on microbial community distances measured using weighted and unweighted UniFrac, Bray-Curtis index, and Jaccard index. *p < 0.05 (FDR adjusted value).

### Microbial communities associated with MRSA, *S. aureus*, and CPS carriage

To determine if MRSA, *S. aureus*, and CPS carriage are associated with differential composition and diversity of the greater microbial community, we used culture-based techniques to determine which human study participants were colonized with CPS, *S. aureus*, and MRSA by nasal and skin swabs and which pets were colonized with CPS, *S. aureus*, and MRSA by nasal and oral swabs. Only 4/30 human subjects were positive for MRSA carriage by these techniques, 1 of which was an index subject presenting with SSTI. Microbial community structure and membership were not significantly different in the axilla/inguinal or nasal sites when comparing MRSA carriers (*n* = 4) to non-carriers (*n* = 26) (Table 3). Results were similar when comparing *S. aureus* carriers (*n* = 4) to non-carriers (*n* = 18) or CPS carriers (*n* = 18) to non-carriers (*n* = 4) among humans, although these comparisons were limited to the 22 participants for whom swabs were available for a second microbial culture using methods harmonized with the animal protocol. In cats, CPS, *S. aureus*, and MRSA carriage in the oral cavity and nares were not associated with significant changes in microbial community composition or diversity. CPS carriage status in dogs was associated with oral cavity microbiota changes, as measured only by the unweighted UniFrac metric (*R* = 0.2495, *p* < 0.05). CPS carriage did not result in dog nasal microbiota differences as determined by the metrics (Table 3). As the majority of CPS cultured from our population of dogs were identified by Polymerase chain reaction (PCR) as *S. pseudintermedius*, with a prevalence rate of 42% among dogs sampled at baseline, we also performed evaluations for *S. pseudintermedius* nasal and oral carriage. However, we did not find any significant associations with microbial community composition or diversity (data not shown). In dogs, *S. aureus* and MRSA carriage in the oral cavity and nares also were not associated with significant changes in microbial community composition or diversity (Table 3).

To further examine microbial interactions in the context of MRSA nasal carriage in humans, we inferred nasal microbiome OTU networks of individuals positive for nasal MRSA carriage (Additional file 5: Figure S2) and individuals negative for nasal MRSA carriage (Additional file 5: Figure S2). Networks were constructed using only OTUs found at greater than 0.1% relative abundance in their respective groups, and only significant interactions (Fisher’s *Z*; *q* = 0.05) are shown. In MRSA-positive individuals, *Staphylococcus* and *Wautersiella* OTUs were negatively correlated (*q* = 0.034; Additional file 5: Figure S2). In MRSA-negative individuals, no OTUs were significantly positively or negatively correlated with *Staphylococcus* OTUs (Additional file 5: Figure S2). However, an unclassified Staphylococcaceae OTU was positively correlated with a number of OTUs including *Lactobacillus*, *Acinetobacter*, *Jeotgalicoccus*, *Psychrobacter*, and *Sphingobacterium*. These findings suggest that OTU interactions may be different depending on MRSA carriage status, but need to be confirmed in larger cohorts.

### Factors that may facilitate sharing of microbiota between humans and pets

**Figure 3:**
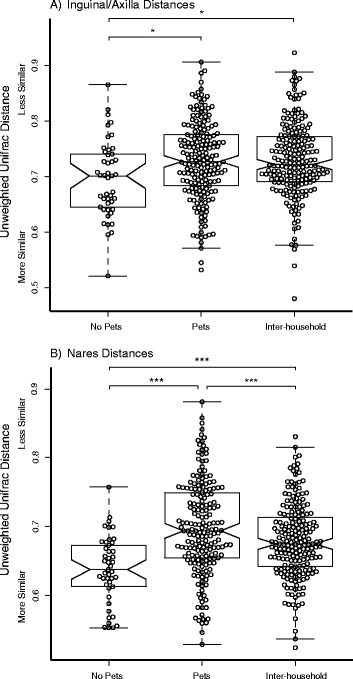
**Effect of pet ownership on sharing of microbiota at human** ***Staphylococcus*** **carriage sites.** Depicted are unweighted UniFrac distances between members of households with pets, members of households without pets, and members of different households (inter-household). The boxes represent the interquartile range (IQR). The whiskers represent the range, excluding outliers. The line in the middle of boxes represents the median. **(A)** Inguinal/axilla distances. **(B)** Nares distances. *p < 0.05, ***p < 0.001 (FDR corrected).

The contact that pets and their owners share can be a factor in the mutual transmission of microbiota. Because previous findings by Song et al. [22] demonstrated that dog ownership increased sharing of microbiota in households, particularly skin microbiota, we analyzed the effect of cohabitation and pet ownership on household microbial community composition at sites of *Staphylococcus* carriage. Pet ownership did not have a significant effect on microbial communities of the nares or the inguinal/axilla microbiota in humans (Table 2). Cohabitating human and pet subjects shared more of their microbiota with their household members than with humans and pets living outside of the household (Table 2). In particular, household had a strong effect on the human nares microbiota, with higher *R* values for beta-diversity metrics that were not weighted for abundance (*R* = 0.49 unweighted UniFrac and *R* = 0.50 binary Jaccard index; both *p* < 0.005), suggesting that rarer OTUs are contributing to differences within the microbial communities observed among households. Unweighted beta-diversity metrics were also significant for inguinal/axilla microbiota, but with lower *R* values (*R* = 0.19 for binary Jaccard index, *p* < 0.01). Household membership similarly was associated with cat and dog microbiota (Table 2), though the greatest effect observed was for cat nares, again using unweighted beta-diversity metrics (*R* = 0.95 for the binary Jaccard index; *p* < 0.005; *R* = 0.75 for the unweighted UniFrac index, *p* < 0.005). When comparing the microbiota of members within the household, people who did not have pets in their households were more similar to each other than people that did have pets in their households, in both the inguinal/axilla regions (*p* < 0.001); Figure 3A) and the nares (*p* < 0.05; Figure 3B).

### The stability of microbiota colonizing staphylococcal carriage sites

To understand how microbiota of staphylococcal carriage sites change over a period of 3 months in cat, dog, and human subjects, we examined differences in the microbiota of subjects present at both study visits. The paired relative abundance graphs are illustrated for individual cat (Additional file 6: Figure S3), dog (Additional file 7: Figure S4), and human (Additional file 8: Figure S5) microbiota at baseline and 3 months, respectively.

The core microbiomes of each host species and anatomical site were determined by identifying OTUs present in at least 90% of host species and anatomical sites at both study visits (Additional file 9: Table S4). To further determine which OTUs were part of the temporally stable microbiomes, we performed Wilcoxon rank-sum tests to determine if those OTUs fluctuated significantly over time. Approximately 12%–15% of the cat oral microbiome was temporally stable, containing OTUs belonging to the families *Porphyromonadaceae*, *Pseudomonadales*, and *Pasteurellaceae*, and class *Clostridiales*. Approximately 13%–19% of the cat nasal microbiome was temporally stable, containing OTUs belonging to the *Pasteurellaceae* and *Moraxellaceae*, and *Staphylococc*us (Additional file 9: Table S4).

Approximately 33%–51% of the core dog nasal microbiome was temporally stable and also contained *Staphylococcus* OTUs. The OTUs that did significantly differ temporally were *Conchiformibius* and unclassified *Pasteurellaceae*, both increasing in relative abundance over the 3-month time period (*p* = 0.012 and *p* = 0.021, respectively). Dog oral cavity temporal core microbiome OTUs consisted of *Porphyromonas*, *Streptococcus*, *Conchiformibius*, and *Pasteurellaceae* and comprised 24% of the microbiota at that site (Additional file 9: Table S4).

Temporally stable OTUs in human healing lesions included *Corynebacterium*, *Micrococcus*, *Staphylococcus*, *Streptococcus*, *Anaerococcus*, and *Finegoldia* but only made up 9%–10% of the colonizing microbiota. In the human nares, *Staphylococcus* and *Streptococcus* OTUs were stable over time and generally comprised 10%–18% of the colonizing microbiota. *Corynebacterium* and *Staphylococcus* OTUs were temporally stable in the inguinal crease/axilla microbiome. However, one *Corynebacterium* OTU in the inguinal crease/axillae samples significantly increased in relative abundance during this time period (*p* = 0.028), but overall, 34%–36% of the colonizing microbiota was temporally stable (Additional file 9: Table S4).

**Figure 4:**
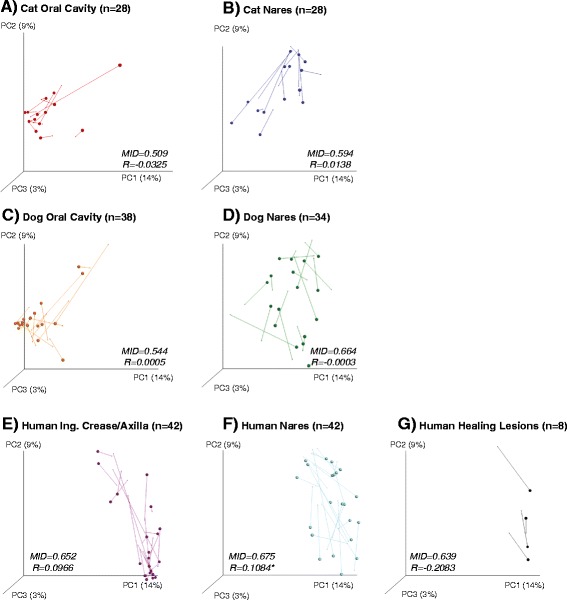
**Unweighted UniFrac 3D PCoA illustrating changes in microbiota over 3 months.** The panels represent microbial community changes over time in the **(A)** cat oral cavity, **(B)** cat nares, **(C)** dog oral cavity, **(D)** dog nares, **(E)** human inguinal crease/axillae, **(F)** human nares, and **(G)** human healing lesions. Each vector represents the microbial community of an individual at baseline and at 3 months, represented by the spheres. The sample number, *n*, is the total sample number from both visits. *MID* median intersample distances. The *R* values are obtained from Table 4; *p < 0.05.

Differences in microbiota over the two time points were also assessed using beta-diversity metrics (Table 4). The cat and dog oral and nasal microbiota did not significantly change by these metrics. Only the human nares and inguinal/axillae temporally changed significantly in microbiota composition by beta-diversity metrics over the 3 months (unweighted UniFrac and binary Jaccard, *p* = 0.02 for all). In the inguinal crease/axillae samples, a *Corynebacterium* OTU varied over time (*p* = 0.03) (Additional file 9: Table S4). Community differences were detected using unweighted beta diversity metrics, indicating that rarer taxa are likely contributing to these shifts. These temporal microbial community shifts, as measured by the unweighted Unifrac and median intersample dissimilarity score, are illustrated in Figure 4. Human subjects in pet-owning households did not significantly differ temporally in their degree of microbial community change at nares or inguinal crease/axillae sites, when compared to non-pet owners.

**Table 4:** **Temporally stability of microbiota at staphylococcal carriage sites**

**Host species**	**Anatomic site**	**Beta diversity metric**	***R*** **statistic and significance**
Cats	Nares	Weighted UniFrac	−0.0623
		Unweighted UniFrac	0.0138
		Bray-Curtis	−0.0260
		Binary Jaccard	0.1084
Cats	Oral cavity	Weighted UniFrac	−0.0566
		Unweighted UniFrac	−0.0325
		Bray-Curtis	−0.0119
		Binary Jaccard	−0.0433
Dogs	Nares	Weighted UniFrac	−0.0190
		Unweighted UniFrac	−0.0003
		Bray-Curtis	−0.0054
		Binary Jaccard	0.0337
Dogs	Oral cavity	Weighted UniFrac	−0.0119
		Unweighted UniFrac	0.0005
		Bray-Curtis	−0.0087
		Binary Jaccard	−0.0624
Humans	Nares	Weighted UniFrac	−0.0223
		Unweighted UniFrac	0.1084*
		Bray-Curtis	0.0309
		Binary Jaccard	0.1180*
Humans	Inguinal/axilla	Weighted UniFrac	−0.0017
		Unweighted UniFrac	0.0966
		Bray-Curtis	0.0336
		Binary Jaccard	0.1962**
Humans	Healing lesions	Weighted UniFrac	−0.1562
		Unweighted UniFrac	−0.2083
		Bray-Curtis	−0.2188
		Binary Jaccard	−0.2396

ANOSIM analyses summary assessing various metadata associations of microbial community distances over two time points using weighted and unweighted UniFrac, Bray-Curtis, and Jaccard indexes. *p < 0.05 and **p < 0.005 (FDR adjusted).

Four of six households with human participants were randomized to treatment, although index participants were sampled at both visits in only three of these households and only two of these households reported compliance with medication usage according to the study decolonization protocol. Mupirocin (nares) and chlorhexidine (skin) decolonization strategy was not correlated with shifts in microbial communities between baseline and 3-month visits, regardless of analysis according to intention-to-treat or analysis as-treated based on participant report of compliance.

To explore the diversity of species within the genus *Staphylococcus* over time, we identified a subset (nares and lesion site only) of archived staphylococcal isolates from people (Additional file 10: Table S5). Overall, *S. epidermidis* was the most commonly identified non-*aureus* staphylococcal isolate, with nasal carriage found in 12 participants (1 index, 11 household members) at the baseline visit, nasal carriage found in 9 participants (2 indexes, 7 household members) at the 3-month visit, and lesion-site contamination found in 3 index participants at the 3-month visit. Other staphylococcal species found at the nares site were (in decreasing order of frequency): *S. haemolyticus* (*n* = 4), *S. cohnii* (*n* = 2), *S. lugdunensis* (*n* = 2), and with *n* = 1 each, *S. capitis*, *S. intermedius*, *S. kloosii*, and *S. warneri*. None of the participants with *S. epidermidis* nasal colonization were nasally colonized with *S. aureus* at the baseline visit. Accounting for correlation of data within person and within household over time, *S. epidermidis* nasal carriage was associated with protection against *S. aureus* nasal carriage (OR 0.19 [95% confidence interval: 0.02, 1.79]), but this estimate of association was not statistically significant (*p* = 0.14). Model results were similar with inclusion of index participant status and decolonization treatment prior to the 3-month visit.

## Discussion

Here, we report the microbiota colonizing humans and companion animals at sites of typical *Staphylococcus* carriage. Pet oral cavity and nasal microbiota were predominantly colonized with Proteobacteria, whereas humans were colonized with greater relative abundances of Firmicutes and Actinobacteria. Upon examination of microbiota colonizing staphylococcal SSTI lesions, we observed a great diversity of microbiota in the lesion and unexpectedly low relative abundance of *Staphylococcus sp*. We did not detect association of microbial communities with MRSA SSTI, MRSA carriage, *S. aureus* carriage, CPS carriage, or human decolonization treatment. We additionally found that humans and pets living in the same household share more microbiota with each other than with human and pets in different households, and pet ownership is associated with diversity of human microbiota within the same household. Finally, we assessed the stability of the microbiota at staphylococcal colonization sites over a 3-month time span and noted that only human microbiota was significantly shifted over time.

Our findings of human microbiota composition are consistent with previously published studies of microbiota colonizing human nares and the inguinal crease/axillae [23-25]. Culture-based analysis of cat and dog oral cavity microbiota previously showed that staphylococci, along with streptococci were among the most prevalent bacteria in pets not exposed to MRSA-carrying humans [26]. The dog oral cavity microbiota, as characterized by 16S rRNA sequencing, revealed many previously unnamed bacterial taxa [27,28]. In our study, these unknown taxa are reflected in the genus-level classifications, with six of the top ten genera being “unclassified”. In a separate study of culture-independent analysis of six healthy canine oral cavities, *Bacteroidetes* was found to be the most abundant phylum [28], while our analyses found that Proteobacteria was the most abundant phylum. Unlike our study and that of Sturgeon et al. [28], Dewhirst et al. did not find *Staphylococcus sp.* to be in the canine oral cavity microbiota, but Firmicutes and Proteobacteria were the most abundant phyla recovered in that study of 51 dogs [27]. Similar to our study, a previous report of the feline oral microbiome found that Proteobacteria was the predominant phylum [29]. Compared to other sites sampled in this study, cat oral cavity was the most diverse and significantly more diverse than the dog oral cavity.

People in households who own pets were colonized with less similar (more diverse) microbiota compared to each other than to people in households without pets. This may indicate enhanced microbial sharing and perhaps more diverse microbial inputs related to pet exposure. Evaluation of the same people in households over time suggests that the magnitude of temporal shifts in microbial communities within host is not strongly influenced by pet ownership. Our results are consistent with a recent study demonstrating that owning a dog increased sharing of microbiota in households, particularly of the skin, and that human skin was colonized with microbiota more similar to the dog living in the household than to other dogs [22]. As Song et al. noted, it is difficult to ascertain which shared human and pet taxa are transient and which have a specific niche. Given recent findings that exposure to pets early in life may be protective of allergic and atopic disease [30,31], it is important to understand the role of pets in transfer and sharing of microbiota, which can influence the immune response [10].

While we have not determined if all pet species are transiently or persistently colonized with *Staphylococcus* bacteria, our data suggest that the colonization is protracted in cats, as at least 90% of the cats at both study visits were nasally colonized with *Staphylococcus*. However, it is important to note that cats may not truly become colonized, as defined by replication of bacteria at the anatomical site, but instead may have frequent nasal exposure to human sources of *Staphylococcus* bacteria through interactive behavior. In our sampling population, owners reported that all cats had at least some contact with people in the house on a daily basis, and the *Staphylococcus* OTUs present in cat nares also belonged to the core microbiota of the human nares, inguinal crease/axilla, and lesion samples. Notably, a limitation of 16S rRNA profiling is that it cannot distinguish between viable and dead bacteria, as the technique relies upon DNA amplification. Therefore, these findings may not represent true colonization but reflect interactive behavior between pets and humans.

The limitations of this study include a small number of households in which humans were enrolled (*n* = 7) and a small proportion of MRSA-positive human subjects (4 of 30 enrolled). Larger sampling populations with comparison groups will be required to conclude whether MRSA colonization influences microbial community composition and diversity. Examining staphylococcal carriage in companion animals, we did not detect any differences in the oral cavity or nasal microbiota that would suggest that CPS, *S. aureus*, or MRSA carriage have an effect on the microbial diversity or composition. Our study was a small case series, which limits the generalizability of our findings. Another limitation is that the V4 region used for sequencing was too short to provide reliable speciation, particularly for *Staphylococcus* bacteria, where several species may have identical or nearly identical sequences in the V4 region. To explore speciation further, we identified staphylococcal species cultured from human nares and lesion site and found that *S. epidermidis* and *S. aureus* were the most common species. Finally, human subjects in our study were randomized to treatment groups, but participant compliance with treatment protocols in this sampled group was poor, which may explain the lack of influence of treatment on shifts in microbial communities at either nares or skin over time.

## Conclusions

We have determined the microbiota in staphylococcal carriage sites of index MRSA patients, their household members, and their mammalian pets over time. Further extension of this work with larger sample sizes will provide insight into a potential modifiable target that may influence MRSA and CPS colonization and infection. We also identified patterns of microbiota transfer between humans and their companion animals, which could have potential implications for recurrent MRSA SSTI, atopic and allergic disease, and other disorders and conditions where environmental microbial transfer may play a role.

## Methods

### Human and animal subject protections

Prior to study initiation, this protocol was reviewed and approved as part of a nested study by the University of Pennsylvania’s Institutional Review Board (protocol 814406) and the Johns Hopkins University Institutional Animal Care and Use Committee (protocol SP10H319).

### Household enrollment

Households were recruited in the context of a study nested within a randomized controlled trial (RCT) enrolling participants with laboratory-confirmed MRSA SSTI and their household members. Eligible index patients presented to an outpatient practice or emergency department of one of five adult and/or pediatric hospitals: hospital 1 is a 782-bed urban adult acute care hospital, hospital 2 is a 500-bed urban adult acute care hospital, hospital 3 is a 300-bed urban adult community hospital, hospital 4 is a 469-bed urban children’s hospital, and hospital 5 is a 551-bed rural adult and pediatric hospital. Prior to enrollment in the study, all index participants underwent individual treatment for their MRSA SSTI under the supervision of the diagnosing physician. All households enrolled in the nested study between July and December of 2012 were eligible to participate in companion animal sampling. All households from enrollment hospitals 1 to 4 between September and December of 2012 were eligible to participate in human sampling.

Two home visits took place at which sampling was conducted for microbiota analysis and culture. At each visit, heads of household were surveyed for household- and pet-related characteristics via interview using an iFormBuilder (iFormBuilder, Herndon, VA) application for iPad (Apple, Cupertino, CA). At the end of the baseline visit, the parent study randomized people (both index participants and household members) at the household level to receive either education (control) or treatment with twice-daily nasal mupirocin ointment for a week, with a body wash at the beginning and end of the week using a 4% chlorhexidine body wash (Hibiclens®, Mölnlycke Health Care, Norcross, Georgia). Treatment was scheduled to occur a few weeks after the baseline visit, and participants completed a diary to indicate their compliance with the protocol. The second home visit took place 3 months after the baseline visit, following treatment of people in households randomized to receive medication. We limited analysis over time to human and companion animal subjects present at both visits, excluding any household members only sampled at the 3-month visit.

### Sample acquisition

The choice of anatomical site for microbiota evaluation was harmonized with sites sampled by the parent study and the RCT in order to compare culture-dependent and culture-independent results. Veterinarians wore freshly laundered scrub jackets and sterile gloves during all sample collection from animals; humans self-sampled. The swabs were stored in 0.3 mL Yeast Cell Lysis Solution (Epicentre Biotechnologies, Madison, WI) at −80°C until DNA extraction.

#### Human sampling

Human subjects were asked to self-sample with Eswabs™ (Copan Diagnostics, Murrieta, CA) for microbiologic culture and Catch-All Sample Collection Swabs (Epicentre Biotechnologies, Madison, WI) for microbial community analysis in nares, pooled axillae/groin, and (for index subjects) at the lesion site. A parent performed the swab sample collection for young children. Self-swabbing has been shown to be an effective and sensitive method for detecting MRSA [32].

#### Animal sampling

Animal subjects were sampled using Sterile BBL™ culturettes (BD, Franklin Lakes, NJ) for microbiologic culture, and Catch-All Sample Collection Swabs (Epicentre Biotechnologies, Madison, WI) for microbial community analysis were used to collect superficial samples from nares and oral cavity, selected for their propensity as *Staphylococcus* carriage sites during a pilot phase of the parent study.

### Culture methods

Eswabs from people were streaked onto BBL CHROMagar MRSA plates (BD Diagnostic Systems, Sparks, MD) to identify MRSA colonization. Phenotypic isolates on the chromogenic media were identified as MRSA, confirmed by the presence of *mecA*.

Culturettes from animals were enriched first in Mueller-Hinton broth + 6.5% NaCl selective for all staphylococci and incubated 16–20 h at 35°C [33]. *MRSA only*: From the overnight incubation, 1 mL of broth was transferred to 9 mL Tryptic Soy Broth + 2.5% NaCl + 3.5 mg/L cefoxitin + 10 mg/L aztreonam to enrich growth of beta-lactam-resistant isolates. From each of these broth enrichment steps, isolates were identified using Columbia CNA agar (*Staphylococcus* selective), then transferred to Baird-Parker agar (selective for coagulase-positive staphylococci (CPS) and designed for *S. aureus*). Phenotypic isolates on Baird-Parker were identified as presumptive CPS. All CPS isolates were tested using a multiplex PCR assay that amplifies species-specific segments of the nuclease gene (*nuc*) for *S. aureus*, in addition to veterinary pathogens *S. pseudintermedius* and *S. schleiferi*, as previously described [34]. Methicillin-resistant isolates (MRSA) were identified by presence of a *mecA/mecC* sequence, with ATCC43300 and LGA251 as *mecA*- and *mecC*-positive controls, respectively [35].

Additionally, Eswabs from people were obtained from the parent study and, following CHROMagar culture, subjected to the same enrichment and identification protocol used for elucidation of animal isolates. Coagulase-negative staphylococcal isolates from the MRSA arm of the enrichment protocol and CPS isolates from both arms of the enrichment protocol (MS- and MR-) were archived and available for additional testing. A subset (nares and lesion site isolates only) of coagulase-negative staphylococcal isolates and CPS isolates not identified through the *nuc* PCR protocol were submitted for species identification and antimicrobial susceptibility testing using the BD Phoenix system (BD Diagnostics, Sparks, MD).

### DNA isolation, amplification, and sequencing of 16S rRNA genes

The Catch-All swab samples were thawed, and 0.5 μL of Ready-Lyse Lysozyme (Epicentre Biotechnologies, Madison, WI) was added to each tube and incubated for 1 h with shaking at 600 rpm and 37°C. The swab was removed, placed into a spin basket, and centrifuged for 1 min at 9,400 × *g* to extract any remaining liquid. The sample was then added to a glass bead tube (0.5 mm; MO BIO, Carlsbad, CA) and vortexed for 10 min at maximum setting. The samples were then incubated in a heat block for 30 min at 65°C and 600 rpm, followed by ice for 5 min and a brief spin. A 150 μL of Protein Precipitation Buffer (Epicentre Biotechnologies, Madison, WI) was added, and the samples were vortexed briefly, then centrifuged at 22,000 × *g* for 10 min. The supernatant was removed and the protein pellet was discarded. The supernatant was mixed with 500 μL isopropanol and inverted to mix. The mixture was added to a spin column from the Genomic DNA Isolation Kit (Life Technologies, Grand Island, NY), and the remaining steps were followed according to manufacturer’s protocol. The samples were eluted with 50 μL Elution Buffer (Life Technologies, Grand Island, NY). For each set of extractions, one blank swab exposed to laboratory air was processed as a negative control.

The V4 region of the 16S rRNA gene was amplified using barcoded primers for the Illumina platform as previously described [36]. Sequencing was performed on the MiSeq instrument (Illumina, San Diego, CA) using 150 base paired-end chemistry at the University of Pennsylvania Next Generation Sequencing Core. As a positive control and for run-to-run quality control, we amplified and sequenced Genomic DNA from Microbial Mock Community B (Even, Low Concentration), v5.1 L, for 16S RNA Gene Sequencing, HM-782D, obtained through BEI Resources, NIAID, and NIH as part of the Human Microbiome Project.

The 16S rDNA sequencing data and metadata generated in this study have been submitted to the NCBI Sequence Read Archive (SRA; http://www.ncbi.nlm.nih.gov/sra/) under Bioproject SRP042152 and accession numbers SRX548769-SRX548772.

### 16S rRNA gene analysis

Paired-end reads were assembled using PANDAseq [37], and quality filtered to include sequences with a *Q* score ≥30. mothur v.1.25.0 [38] was employed to remove sequences <248 bp and >255 bp in length and sequences with homopolymers >10 bp in length. QIIME v. 1.6 [39] was used for further downstream processing and analyses. OTUs were defined using 97% sequence similarity with CD-HIT [40,41], and a representative sequence from each OTU containing ≥10 sequences was chosen for downstream analyses (based on the most abundant sequence). Chimeric sequences were removed using ChimeraSlayer [42]. Representative sequences were assigned to genera using the Ribosomal Database Project (RDP) classifier v 2.2 [43], multiple sequence alignment was performed using PyNAST [44], and a phylogeny was built with FastTree [45]. The samples were rarified to 10,000 sequences per sample for calculating alpha- and beta-diversity metrics.

### Bioinformatics quality assurance

For quality control purposes, water and processed blank samples were sequenced and analyzed through the bioinformatics pipeline. Taxa that were present in the water or laboratory air blank samples at >4 standard deviations above the mean when compared to the other sample types were removed, in addition to sequences identified as cyanobacteria or ‘unclassified’ (Additional file 11: Table S6). For further quality control assurance and to ensure run-to-run reproducibility, genomic DNA from Microbial Mock Community B (BEI Resources) was sequenced and the expected sequences were compared to the obtained sequences. Published best practices were used as guidelines [46].

### Statistics

Alpha diversity and taxonomical relative abundances are reported as the median with standard deviation. *P* values were calculated using the Wilcoxon rank-sum test and adjusted for multiple comparisons using the false discovery rate (FDR) correction. Statistical tests were run in R v. 3.0.3 [47]. To determine which factors were most important in determining microbial composition, statistical tests were performed using the non-parametric analysis of similarities (ANOSIM) with four different distance metrics [48]: weighted UniFrac, unweighted UniFrac, Bray-Curtis, and binary Jaccard. Additional risk factor analyses were performed using unadjusted and adjusted logistic regression analysis, accounting for correlation within subject and household, in Stata 13 (StataCorp, College Station, TX). Network analyses comparing OTU interactions in MRSA carriers to non-carriers were conducted using the CoNet plugin [49] in Cytoscape [50]. Spearman rank correlation coefficients were used to calculate positive and negative interactions, and significance of interactions was tested using the Fisher’s *Z* transformation and a *p* value threshold of 0.05, corrected for multiple testing using the Benjamini-Hochberg method. Only significant interactions were included in the network.

## Availability of supporting data

The data sets supporting the results of this article are included with the article and its additional files.

## Additional files

Additional file 1: Table S1. Sequence counts per host species and site ID. The mean, media, and range of sequences obtained by Illumina 16S rRNA sequencing are listed. Additional file 2: Figure S1. The genus-level “pocket pet” nasal planum (NP) and oral cavity (OC) microbiota. Microbiota of the four “pocket pet” species are shown with the top 25 pocket pet microbiome genera. **A)** Hamster oral cavities and nasal plana, **B)** ferret oral cavity and nasal planum, **C)** rabbit oral cavity and nasal planum, and **D)** sugar glider oral cavity/nasal planum combined sample. Additional file 3: Table S2. Genus-level relative abundance. A complete genus-level relative abundance table of all samples. Additional file 4: Table S3. Core microbiomes. Core OTUs present in each sampled host species and anatomical site. OTUs present in 90% of samples are in non-bold text; OTUs present in 100% of samples are in bold and italic text. Additional file 5: Figure S2. OTU network inference of nasal microbiomes according to MRSA carrier status. Network connectivities of OTUs present at greater than 0.1% relative abundance in MRSA carriers (*n* = 4) **(A)** and in MRSA non-carriers (*n* = 22) **(B)**. The red lines represent a negative correlation (mutual exclusion) and the green lines represent a positive correlation (co-present) as calculated by Spearman rank correlation coefficients. Additional file 6: Figure S3. Relative abundance charts depicting site-specific feline microbiota over two visits, 3 months apart. **A)** Cat oral cavity; **B)** cat nares. The numbers on the *x*-axis correspond to the study subject number. The top ten median taxa for each sample site are used in the legend. Additional file 7: Figure S4. Relative abundance charts depicting canine site-specific microbiota over two visits, 3 months apart. **A)** Dog oral cavity; **B)** dog nares. The numbers on the *x*-axis correspond to the study subject number. The top ten median taxa for each sample site are used in the legend. Additional file 8: Figure S5. Relative abundance charts depicting human site-specific microbiota over two visits, 3 months apart. **A)** Human inguinal crease/axillae, **B)** human nares, and **C)** healing human lesions. The numbers on the *x*-axis correspond to the study subject number. The top ten median taxa for each sample site are used in the legend. Additional file 9: Table S4. Core microbiomes over two visits, 3 months apart. Taxa present in 90% samples at both visits 1 and 2. Taxa present in 100% of samples at visit 1 and visit 2 are denoted with bold italics. Median relative abundance levels are reported, and a Wilcoxon rank-sum tests were conducted to assess if taxa levels were significantly different between the two time visits. The total core compositions at baseline and the 3-month visit are additionally reported. Additional file 10: Table S5. Species identification using the BD Phoenix system of cultured non-*aureus* isolates in the genus *Staphylococcus* from human nares and lesions according to visit. The section sign (§) indicates participants living in households without pets. Dagger (†) indicates participants at the 3-month visit who reported compliance with decolonization treatment between baseline and 3-month visits. Additional file 11: Table S6. Putative contaminants removed from 16S rRNA sequence analysis. The taxa that were removed due to their higher abundance (4 standard deviations above the mean) in water and blank processed swabs compared to pet and human samples.

## Abbreviations

MRSA: Methicillin-resistant *Staphylococcus aureus*
CA-MRSA: Community-associated MRSA
HA-MRSA: Hospital-associated MRSA
OTU: Operational taxonomic unit
PCR: Polymerase chain reaction
16S rRNA gene: 16S ribosomal ribonucleic acid gene
CPS: Coagulase-positive *Staphylococcus*
PCoA: Principal coordinates analysis
