# Supplementary material for: The shared microbiota of humans and companion animals as evaluated from Staphylococcus carriage sites
Source: Microbiome. 2015 Jan 23;3:2. doi: 10.1186/s40168-014-0052-7 (PMC4335418; doi:10.1186/s40168-014-0052-7)
Supplement: Additional file 1: Table S1. — Sequence counts per host species and site ID. The mean, media, and range of sequences obtained by Illumina 16S rRNA sequencing are listed. [file 40168_2014_52_MOESM1_ESM.docx]

| **Host and Site ID** | **Mean** | **Median** | **Range** |
| --- | --- | --- | --- |
| Cat Mouth | 48476.55882 | 40417.5 | 24033-106105 |
| Cat Nose | 32791.70588 | 25759.5 | 11995-82064 |
| Dog Mouth | 43789.52727 | 41015 | 19705-78840 |
| Dog Nose | 39422.15094 | 36230 | 17232-94242 |
| Human Inguinal/Ax | 37758.41176 | 32977 | 10752-97916 |
| Human Nose | 37399.82353 | 32428 | 12682-92416 |
| Human Lesion | 38047.6 | 34513.5 | 11455-105605 |

**Additional Table 1: Sequence counts per host species and site ID.** The mean, media, and range of sequences obtained by Illumina 16S rRNA sequencing are listed.
