# Supplementary figures and images for: The shared microbiota of humans and companion animals as evaluated from Staphylococcus carriage sites
Source: Microbiome. 2015 Jan 23;3:2. doi: 10.1186/s40168-014-0052-7 (PMC4335418; doi:10.1186/s40168-014-0052-7)

# **A) Hamsters (n=4)**

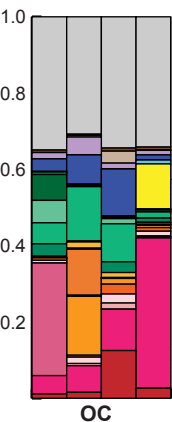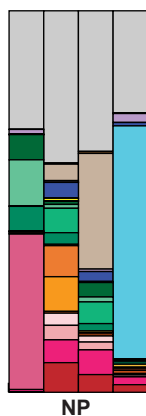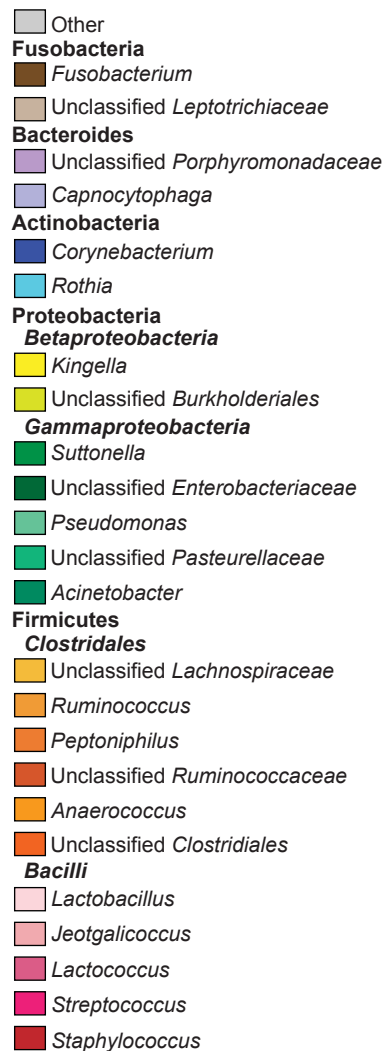

# **B) Ferret (n=1)**

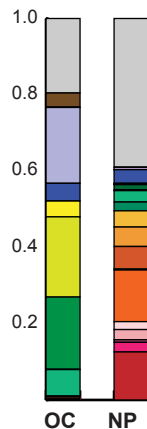

# **C) Rabbit (n=1)**

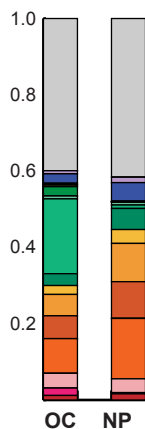

# **D) Sugarglider (n=1)**

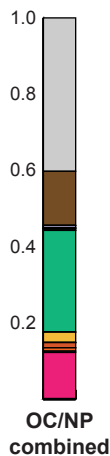

Supplement: Additional file 2: Figure S1. — The genus-level “pocket pet” nasal planum (NP) and oral cavity (OC) microbiota. Microbiota of the four “pocket pet” species are shown with the top 25 pocket pet microbiome genera. A) Hamster oral cavities and nasal plana, B) ferret oral cavity and nasal planum, C) rabbit oral cavity and nasal planum, and D) sugar glider oral cavity/nasal planum combined sample. [file 40168_2014_52_MOESM2_ESM.pdf]

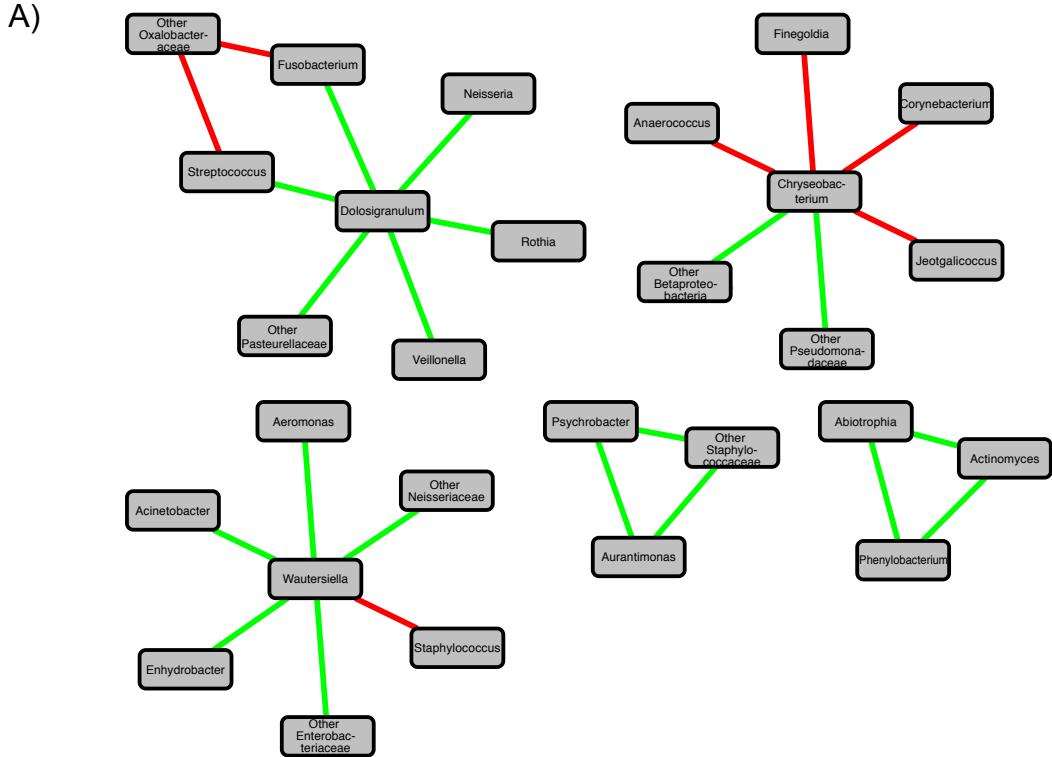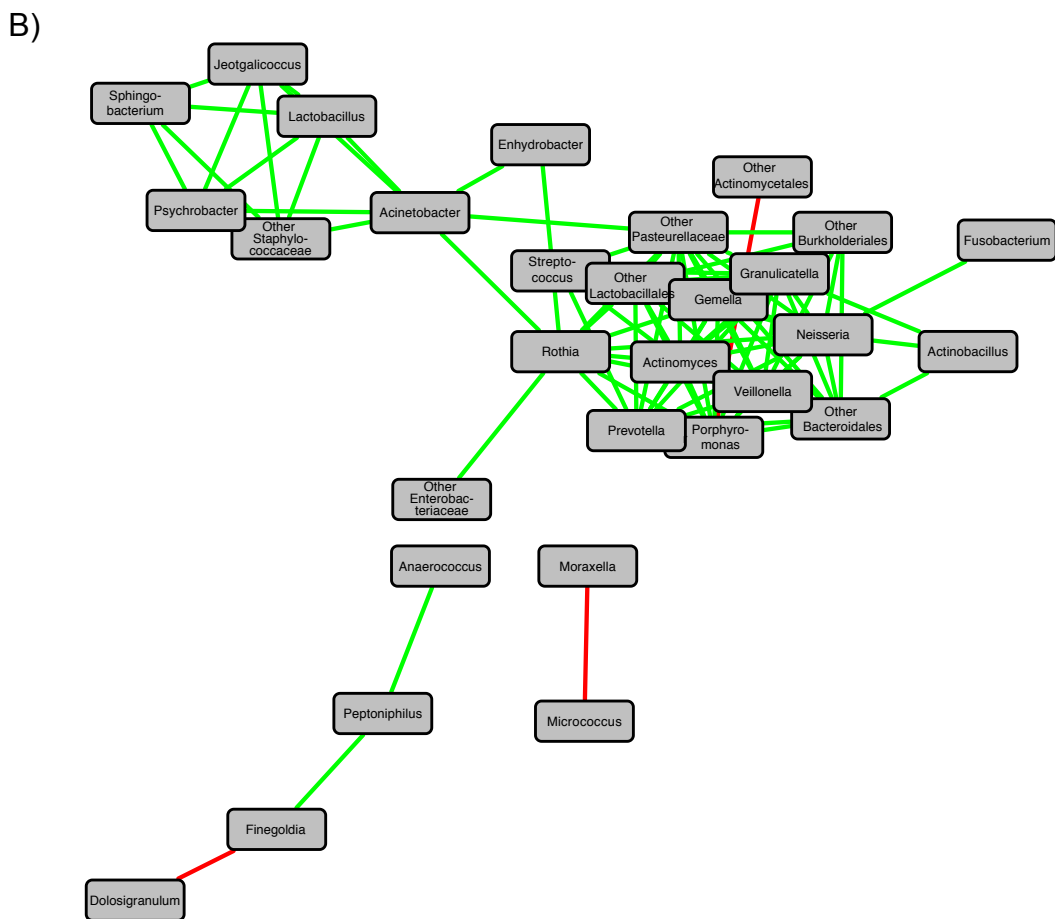

Supplement: Additional file 5: Figure S2. — OTU network inference of nasal microbiomes according to MRSA carrier status. Network connectivities of OTUs present at greater than 0.1% relative abundance in MRSA carriers (n = 4) (A) and in MRSA non-carriers (n = 22) (B). The red lines represent a negative correlation (mutual exclusion) and the green lines represent a positive correlation (co-present) as calculated by Spearman rank correlation coefficients. [file 40168_2014_52_MOESM5_ESM.pdf]

A) Cat Oral Cavity

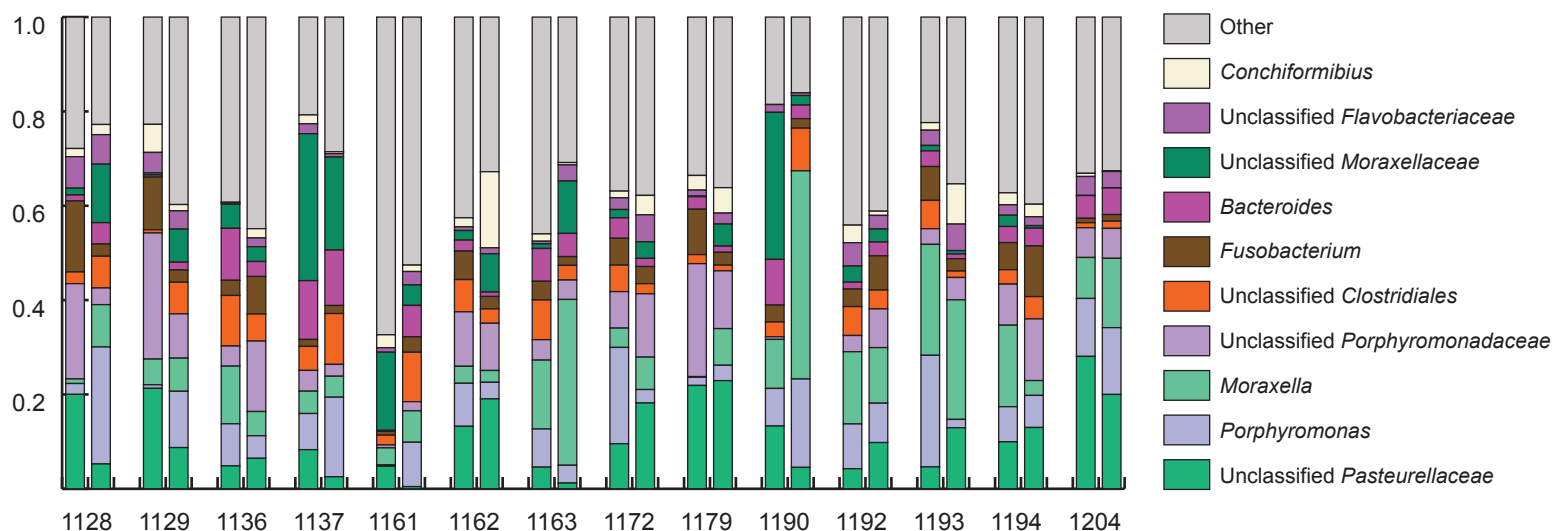

B) Cat Nares

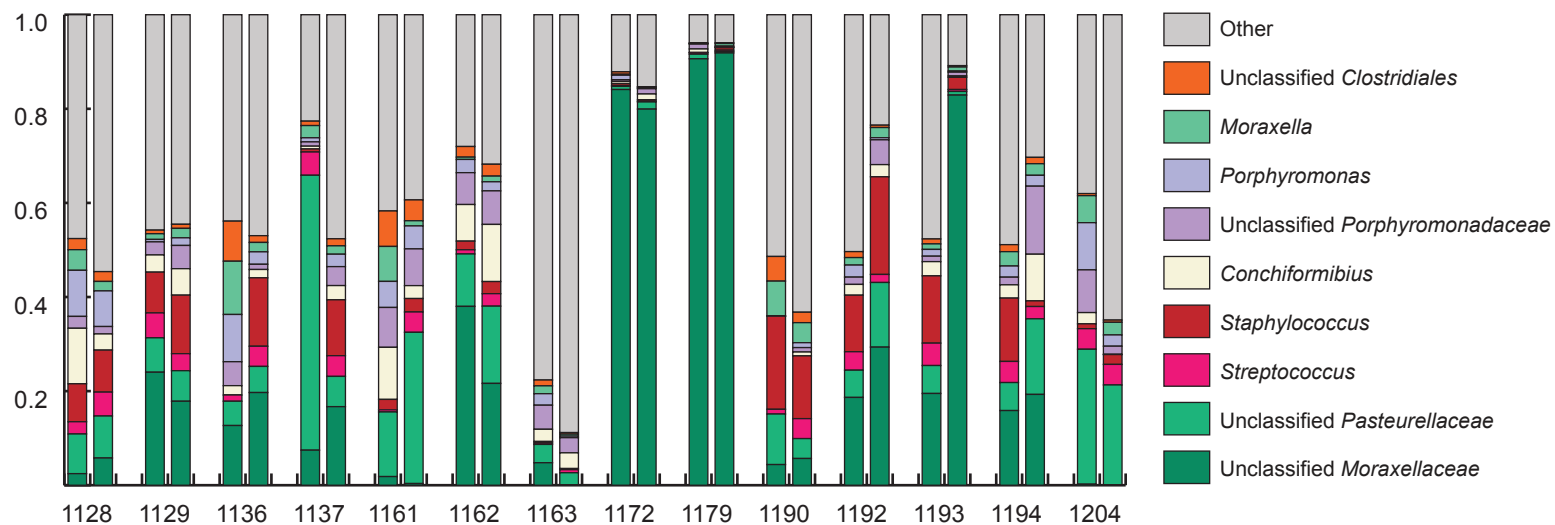

Supplement: Additional file 6: Figure S3. — Relative abundance charts depicting site-specific feline microbiota over two visits, 3 months apart. A) Cat oral cavity; B) cat nares. The numbers on the x-axis correspond to the study subject number. The top ten median taxa for each sample site are used in the legend. [file 40168_2014_52_MOESM6_ESM.pdf]

A) Dog Oral Cavity

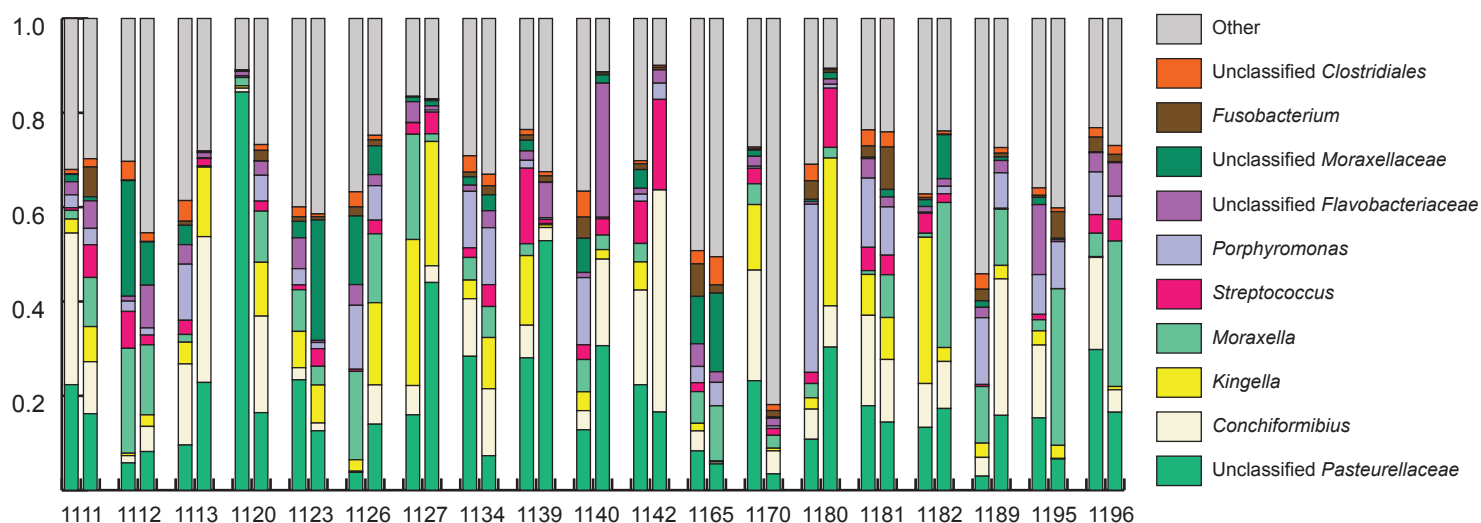

B) Dog Nares

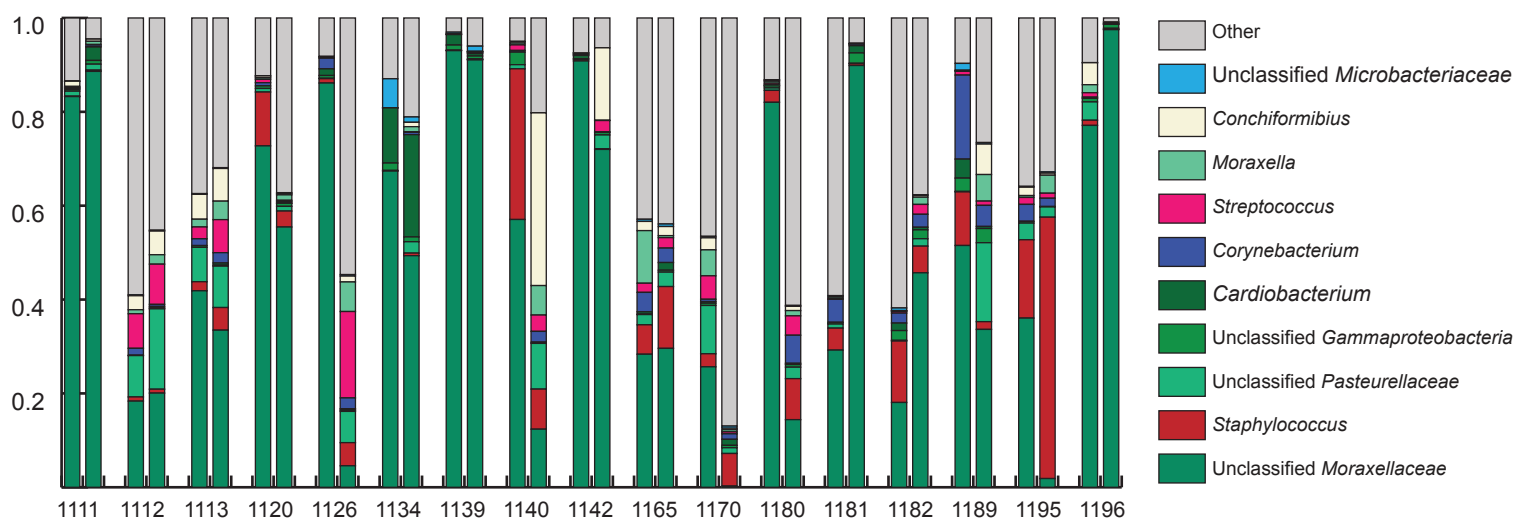

Supplement: Additional file 7: Figure S4. — Relative abundance charts depicting canine site-specific microbiota over two visits, 3 months apart. A) Dog oral cavity; B) dog nares. The numbers on the x-axis correspond to the study subject number. The top ten median taxa for each sample site are used in the legend. [file 40168_2014_52_MOESM7_ESM.pdf]

A) Human Inguinal Crease/Axilla

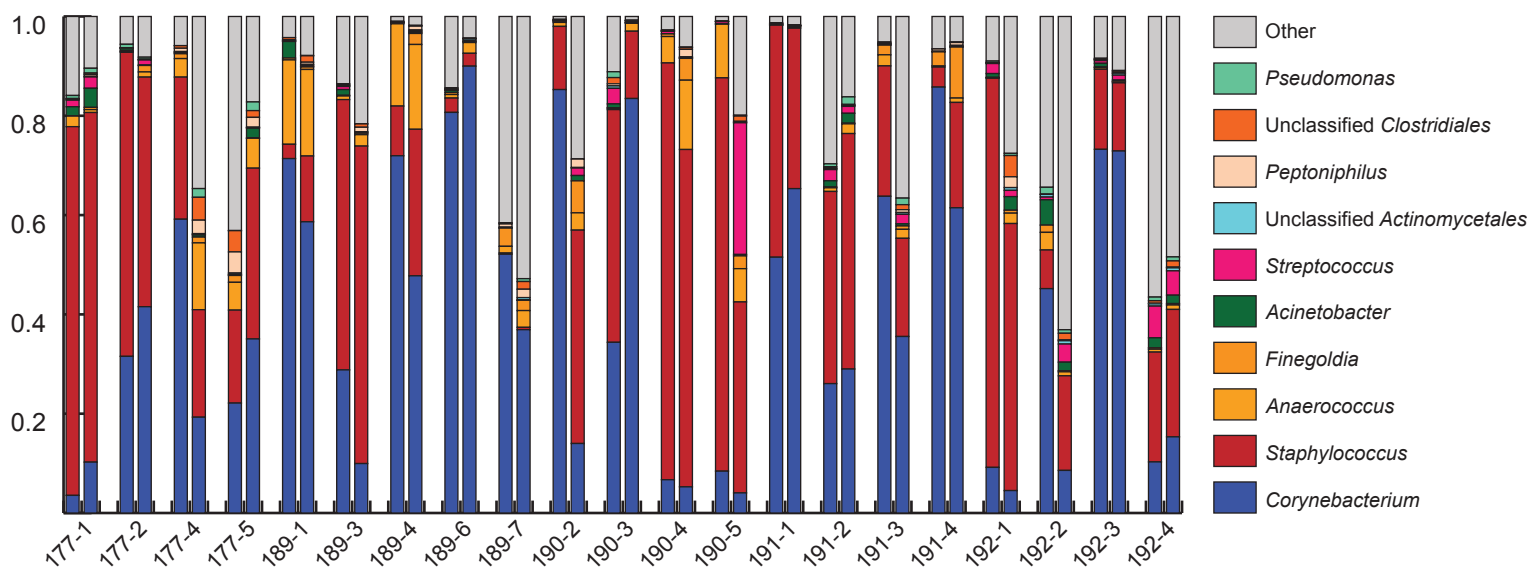

B) Human Nares

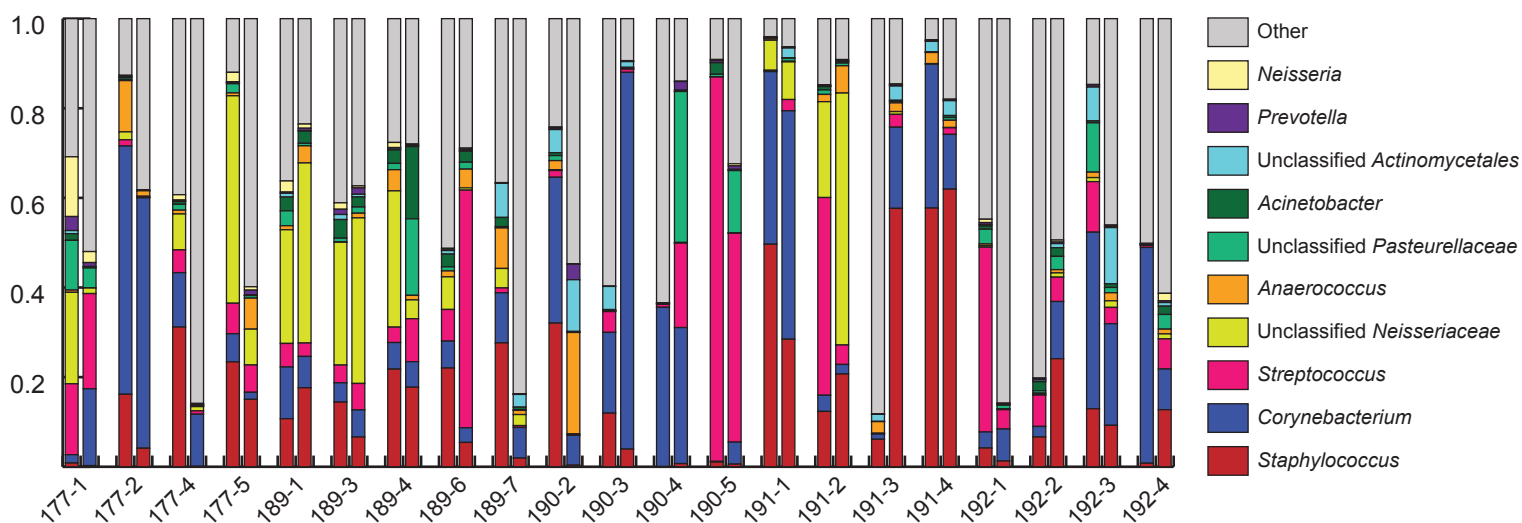

C) Human Healing Lesions

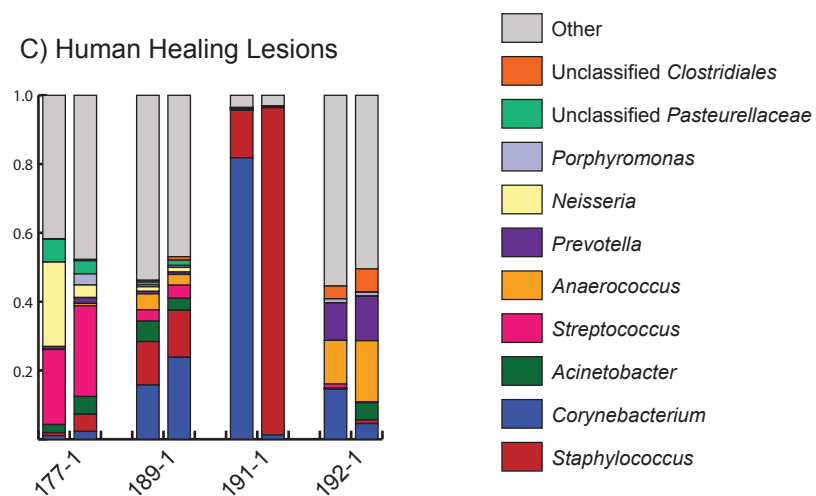

Supplement: Additional file 8: Figure S5. — Relative abundance charts depicting human site-specific microbiota over two visits, 3 months apart. A) Human inguinal crease/axillae, B) human nares, and C) healing human lesions. The numbers on the x-axis correspond to the study subject number. The top ten median taxa for each sample site are used in the legend. [file 40168_2014_52_MOESM8_ESM.pdf]
