# Supplementary material for: The shared microbiota of humans and companion animals as evaluated from Staphylococcus carriage sites
Source: Microbiome. 2015 Jan 23;3:2. doi: 10.1186/s40168-014-0052-7 (PMC4335418; doi:10.1186/s40168-014-0052-7)
Supplement: Additional file 10: Table S5. — Species identification using the BD Phoenix system of cultured non-aureus isolates in the genus Staphylococcus from human nares and lesions according to visit. The section sign (§) indicates participants living in households without pets. Dagger (†) indicates participants at the 3-month visit who reported compliance with decolonization treatment between baseline and 3-month visits. [file 40168_2014_52_MOESM10_ESM.docx]

|  | **Baseline visit** | **Three-month visit** |
| --- | --- | --- |
| Index participant nares | *S. epidermidis* (*n*=1)  *S. haemolyticus* (*n*=1)  *S. warneri* (*n*=1) | *S. epidermidis* (*n*=1†, *n*=1§)  *S. haemolyticus* (*n*=1)  *S. kloosii* (n=1†) |
| Index participant lesion site | No *Staphylococcus sp.* cultured | *S. epidermidis* (*n*=1, *n*=1†, *n*=1§) |
| Household member nares | *S. capitis* (*n*=1)  *S. epidermidis* (*n*=10, *n*=1§)  *S. lugdunensis* (*n*=1) | *S. cohnii* (*n*=2†)  *S. epidermidis* (*n*=1, *n*=3†, *n*=3§)  *S. haemolyticus* (*n*=1, *n*=1†)  *S. intermedius* (*n*=1†)  *S. lugdunensis* (*n*=1§) |

**Additional Table 6:** Species identification using the BD Phoenix system of cultured non-*aureus* isolates in the genus *Staphylococcus* from human nares and lesions according to visit (§ indicates participants living in households without pets; † indicates participants at the three-month visit who reported compliance with decolonization treatment between baseline and three-month visits).
