# Supplementary material for: The shared microbiota of humans and companion animals as evaluated from Staphylococcus carriage sites
Source: Microbiome. 2015 Jan 23;3:2. doi: 10.1186/s40168-014-0052-7 (PMC4335418; doi:10.1186/s40168-014-0052-7)
Supplement: Additional file 11: Table S6. — Putative contaminants removed from 16S rRNA sequence analysis. The taxa that were removed due to their higher abundance (4 standard deviations above the mean) in water and blank processed swabs compared to pet and human samples. [file 40168_2014_52_MOESM11_ESM.docx]

**Additional Table 5: Putative contaminants removed from 16S rRNA sequence analysis.** The taxa that were removed due to their higher abundance (4 standard deviations above the mean) in water and blank processed swabs compared to pet and human samples.

**Mock Swab Contaminants**

Bacteria;Actinobacteria;Actinobacteria;Actinomycetales;Streptomycetaceae;Streptomyces

Bacteria;Chlamydiae;Chlamydiae;Chlamydiales;Parachlamydiaceae;Parachlamydia

Bacteria;Deinococcus-Thermus;Deinococci;Deinococcales;Trueperaceae;Truepera

Bacteria;Firmicutes;Bacilli;Bacillales;Paenibacillaceae;Brevibacillus

Bacteria;Firmicutes;Bacilli;Bacillales;Paenibacillaceae;Other

Bacteria;Firmicutes;Bacilli;Bacillales;Paenibacillaceae;Paenibacillus

Bacteria;Fusobacteria;Fusobacteria;Fusobacteriales;Leptotrichiaceae;Sneathia

Bacteria;Nitrospira;Nitrospira;Nitrospirales;Nitrospiraceae;Nitrospira

Bacteria;Proteobacteria;Alphaproteobacteria;Rhizobiales;Beijerinckiaceae;Chelatococcus

Bacteria;Proteobacteria;Alphaproteobacteria;Rhizobiales;Bradyrhizobiaceae;Bosea

Bacteria;Proteobacteria;Alphaproteobacteria;Rhizobiales;Hyphomicrobiaceae;Devosia

Bacteria;Proteobacteria;Alphaproteobacteria;Sphingomonadales;Erythrobacteraceae;Erythrobacter

Bacteria;Proteobacteria;Alphaproteobacteria;Sphingomonadales;Erythrobacteraceae;Other

Bacteria;Proteobacteria;Alphaproteobacteria;Sphingomonadales;Sphingomonadaceae;Novosphingobium

Bacteria;Proteobacteria;Alphaproteobacteria;Sphingomonadales;Sphingomonadaceae;Sphingopyxis

Bacteria;Proteobacteria;Betaproteobacteria;Burkholderiales;Burkholderiales_incertae_sedis;Leptothrix

Bacteria;Proteobacteria;Betaproteobacteria;Burkholderiales;Comamonadaceae;Roseateles

Bacteria;Proteobacteria;Betaproteobacteria;Burkholderiales;Comamonadaceae;Rhodoferax

Bacteria;Proteobacteria;Betaproteobacteria;Burkholderiales;Oxalobacteraceae;Herbaspirillum

Bacteria;Proteobacteria;Betaproteobacteria;Burkholderiales;Oxalobacteraceae;Duganella

Bacteria;Proteobacteria;Betaproteobacteria;Burkholderiales;Oxalobacteraceae;Herminiimonas

Bacteria;Proteobacteria;Betaproteobacteria;Hydrogenophilales;Hydrogenophilaceae;Hydrogenophilus

Bacteria;Proteobacteria;Betaproteobacteria;Rhodocyclales;Rhodocyclaceae;Shinella

Bacteria;Proteobacteria;Gammaproteobacteria;Enterobacteriales;Enterobacteriaceae;Citrobacter

Bacteria;Proteobacteria;Gammaproteobacteria;Enterobacteriales;Enterobacteriaceae;Pantoea

Bacteria;Proteobacteria;Gammaproteobacteria;Enterobacteriales;Enterobacteriaceae;Serratia

Bacteria;Proteobacteria;Gammaproteobacteria;Salinisphaerales;Salinisphaeraceae;Salinisphaera

Bacteria;Proteobacteria;Gammaproteobacteria;Thiotrichales;Piscirickettsiaceae;Methylophaga

Bacteria;Proteobacteria;Gammaproteobacteria;Xanthomonadales;Xanthomonadaceae;Stenotrophomonas

**Mock PCR Contaminants**

Bacteria;Acidobacteria;Acidobacteria_Gp1;Gp1;Other;Other

Bacteria;Acidobacteria;Acidobacteria_Gp16;Gp16;Other;Other

Bacteria;Actinobacteria;Actinobacteria;Actinomycetales;Geodermatophilaceae;Other

Bacteria;Proteobacteria;Alphaproteobacteria;Caulobacterales;Caulobacteraceae;Brevundimonas

Bacteria;Proteobacteria;Alphaproteobacteria;Rhizobiales;Bradyrhizobiaceae;Bradyrhizobium

Bacteria;Proteobacteria;Alphaproteobacteria;Rhizobiales;Bradyrhizobiaceae;Other

Bacteria;Proteobacteria;Alphaproteobacteria;Rhodobacterales;Rhodobacteraceae;Other

Bacteria;Proteobacteria;Alphaproteobacteria;Rhodospirillales;Acetobacteraceae;Other

Bacteria;Proteobacteria;Alphaproteobacteria;Sphingomonadales;Sphingomonadaceae;Sphingomonas

Bacteria;Proteobacteria;Betaproteobacteria;Burkholderiales;Alcaligenaceae;Alcaligenes

Bacteria;Proteobacteria;Betaproteobacteria;Burkholderiales;Burkholderiaceae;Burkholderia

Bacteria;Proteobacteria;Betaproteobacteria;Burkholderiales;Burkholderiaceae;Other

Bacteria;Proteobacteria;Betaproteobacteria;Burkholderiales;Comamonadaceae;Pelomonas

Bacteria;Proteobacteria;Deltaproteobacteria;Myxococcales;Cystobacteraceae;Other

Bacteria;Proteobacteria;Deltaproteobacteria;Myxococcales;Other;Other
